# COVID-19 after the first wave of the pandemic among employees from a German university hospital: prevalence and questionnaire data

**DOI:** 10.25122/jml-2022-0126

**Published:** 2022-09

**Authors:** Anna Catharina Sellmeier, Andreas Elsner, Tim Niedergassel, Johannes Schmitz, Sebastian Rehberg, Claudia Hornberg, Thomas Vordemvenne, Dirk Wähnert

**Affiliations:** 1 Department of Trauma Surgery and Orthopedics, University Hospital OWL of Bielefeld University, Bielefeld, Germany; 2 DIOOS – German Institute for Orthopedics, Osteopathy and Sports Medicine, Bielefeld, Germany; 3 MVZ Medicum Brake, Bielefeld, Germany; 4 Faculty of Business and Economics, University of Applied Sciences, Neu-Ulm, Germany; 5 Department of Anaesthesiology, Intensive Care and Emergency Medicine, University Hospital OWL of Bielefeld University, Bielefeld, Germany; 6 Medical Faculty, University Bielefeld, Bielefeld, Germany

**Keywords:** SARS-CoV-2, health care workers, antibody

## Abstract

The SARS-CoV-2 pandemic has changed lives around the world. In particular, healthcare workers faced significant challenges as a result of the pandemic. This study investigates the seroprevalence of SARS-CoV-2 in March–April 2020 in Germany among healthcare workers and relates it to questionnaire data. In June 2020, all employees of the reporting hospital were offered a free SARS-CoV-2 antibody test. The first 2,550 test results were sent along with study documents. The response rate was 15.1%. The COVID-19 PCR test prevalence amongst health care workers in this study was 1.04% (95% CI 0.41–2.65%), higher by a factor of 5 than in the general population (p=0.01). The ratio of seroprevalence to PCR prevalence was 1.5. COVID-19-associated symptoms were also prevalent in the non-COVID-19-positive population. Only two symptoms showed statistically significant odds ratios, loss of smell and loss of taste. Health care workers largely supported non-pharmaceutical interventions during the initial lockdown (93%). Individual behavior correlated significantly with attitudes toward policy interventions and perceived individual risk factors. Our data suggest that healthcare workers may be at higher risk of infection. Therefore, a discussion about prioritizing vaccination makes sense. They also support offering increased SARS-CoV-2 testing to hospital workers. It is concluded that easier access to SARS-CoV-2 testing reduces the number of unreported cases. Furthermore, individual attitudes toward rules and regulations on COVID-19 critically influence compliance. Thus, one goal of public policy should be to maintain high levels of support for non-pharmaceutical interventions to keep actual compliance high.

## INTRODUCTION

In January 2020, the SARS-CoV-2 virus reached Germany [1]. By March 2020, the first pandemic wave brought coronavirus disease (COVID-19) to more than 100 countries worldwide [2]. Test methods were rapidly developed to identify and isolate infected individuals. However, reports of silent infections increased [1, 3]. The affected individuals had no or only mild symptoms, so testing for SARS-CoV-2 was not done due to limited capacity. Nevertheless, these individuals could spread the infection. As of April 2020, according to the Robert Koch Institute, information on the incidence of COVID-19 was still relatively unknown [4]. It was assumed that the number of infected individuals who actually became ill ranged from 51% to 81%. This means that 19–49% of infected persons do not show any symptoms [4]. Furthermore, it was assumed that only a part of the infected/sick persons would be recorded. The actual number of infected persons can only be estimated. In China at the time, the number of reported infections was assumed to be merely 5–9% of total infections, so the total number of infections was greater by a factor of 11 to 20 than the number of reported cases [4]. These circumstances and limited knowledge about the virus, the infection routes, and the disease increased the insecurity in the population, especially amongst medical personnel who treated those infected.

Since its onset, the Corona pandemic has impacted all medical sectors, from pre-hospital rescue to rehabilitation. Specifically, inpatient care has been limited, especially in the selective sector. In this regard, during the first wave, there were mainly local clusters of COVID-19 cases (hot spots). Our own studies show a double burden on individual hospitals resulting from the care of COVID-19 patients and an accumulation of emergency patients [5]. This double burden primarily affected maximum-care hospitals.

The development and introduction of antibody testing in May 2020 allowed testing for SARS-CoV-2 infections. At that time, there were more than 180,000 officially confirmed SARS-CoV-2 infections in Germany since the beginning of the pandemic and more than 38,000 in North Rhine-Westphalia [6]. The maximum incidence during the first wave in Germany was 43, the hospitalization rate was 15%, and the rate of patients requiring intensive care was approximately 5%.

**Figure 1:**
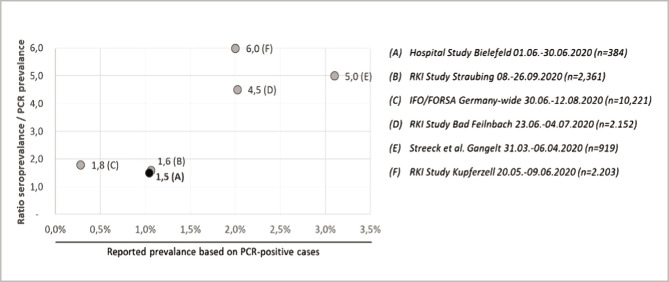
Ratio of antibody positives vs. PCR positives in different studies in Germany in or after the first and before the second wave (April to September 2020).

The type of test performed also has a significant impact on the infections detected. The ratio of seroprevalence to PCR prevalence found in five studies between April and September 2020 with over 17,000 participants ranged from 1.6 to 6.0 (Figure 1).

The aim of the present study was to investigate the: (a) PCR-confirmed prevalence in the population of healthcare workers compared to the general population, (b) the ratio between cases identified by positive antibody tests *vs*. reported PCR-confirmed cases, (c) prevalence of symptoms associated with COVID-19 and (d) behavior and attitude differences between health care workers and the general population as part of an investigation into potential risk factors for COVID-19.

## MATERIAL AND METHODS

### Participant recruitment

Beginning in June 2020, all hospital employees were offered free SARS-CoV-2 antibody testing by the hospital's own occupational health service (inclusion criterion). All participants were healthy at the time of participation. Test results were mailed through the occupational health department. The first 2,550 results (June and July 2020) were mailed along with a study information letter, consent form, and questionnaire (Supplementary Figure 1). This approach would have allowed approximately 50% of all hospital employees to complete a questionnaire. Previous PCR test results and antibody test results were self-reported. Employees who wished to participate in the study returned the signed informed consent form and completed questionnaire to the study center in the enclosed envelope. The returned documents were checked at the study center and pseudonymized if complete (informed consent).

This study was approved by the responsible review board, and all methods were performed in accordance with the relevant guidelines and regulations.

### COVID-19 antibody testing

For this study, the total antibody test from Roche (Elecsys^®^, Basel, Switzerland) was used (predominantly immunoglobulin (Ig)G, but also IgM). It is an electrochemiluminescence immunoassay (E-CLIA). This test has a specificity of 99.8% and, if used more than 14 days after a positive PCR test, a sensitivity of 100%.

### Questionnaire

The questionnaire (in German) is included as supplementary material.

The questionnaire consisted of 26 main items on various topics. Questions were asked about personal background (*e.g*., age, gender, migration background, education, occupation, housing situation), health (*e.g*., diseases, risk factors, symptoms of COVID-19), behavior, and private/professional contacts during the first lockdown period. The questions on attitude towards political measures and perceived personal risks were taken from representative studies on the general population by the German Federal Institute for Risk Assessment. For the other questions, no specific validation was performed.

### Data handling and statistical methods

The paper-based questionnaires were transferred to Microsoft Excel 2016 (Version 16.16.1, Microsoft Cooperation, Redmond, USA). In order to check for data entry errors, 10% of the questionnaires were entered twice. No data entry errors were found so that that full double text entry could be dispensed with. The data were categorized for statistical processing. Statistical evaluation was done using Microsoft Excel and the SPSS software package (Version 25, SPSS, Chicago, IL, USA).

The share of positive PCR and antibody tests was compared to the officially reported prevalence data for the region, which was published by the Robert Koch Institute (RKI). Regional data were adjusted for the age and sex distribution of the study population. The number of positive antibody test results was adjusted for test specificity (99.8%). The ratio of antibody prevalence to PCR test prevalence was compared to results from other studies in the German population during the same time period before the second wave of the pandemic in Germany (*i.e*., before October 2020).

Statistical testing was done using a binomial model. Wilson confidence intervals were used for prevalence estimates (due to the low prevalence estimates) and Jeffrey's confidence interval for the ratio between the total number of positive cases (including antibody positive cases) and reported PCR positive cases (due to low n) [7]. P values for tests concerning prevalence and symptoms were adjusted using the Bonferroni-Holm correction [8].

Questions on attitude towards political measures and perceived personal risks were compared to representative studies on the general population by the German Federal Institute for Risk Assessment (BfR). The level of association amongst variables on attitude, health background, and behavior within the study population was done using the chi-square test with significance set at p<0.05. As the data set for the analyses on attitude, risk factors, and behavior was independent from the data on positive test rates, p values in these analyses were separately adjusted using the Bonferroni-Holm correction again for multiple tests.

## RESULTS

Of the 2,550 questionnaires sent, 384 were completed and returned to the study center (response rate of 15.1%). 301 participants were female (78.4%) and 83 male (21.6%). All participants were of working age (range from 19 to 66 years) with a mean of 45.6 years (SD 12.3) and similar age distribution for women (45.5 years, SD 12.4) and men (46.3 years, SD 11.6). 297 (77.3%) of the 384 participants worked in a medical profession. With these characteristics, a representative population of the healthcare workers within the hospital was evaluated. 360 (93.8%) participants were fully at work during the first lockdown in March/April 2020. 97 (25.3%) participants stated that they had been abroad since December 2019. Further details on the study population are shown in Table 1. Supplementary Table 1 shows a summary of the chi-square test results.

**Table 1:** Characteristics of the study population. HCW – healthcare worker.

Study population characteristics (n=384)				
	Mean	Standard deviation	Min	Max
**Age – all**	45.64	12.26	19	66
**Age – neg**.	45.74	12.20	20	66
**Age – pos**.	39.67	14.83	19	57
**People in household – all**	2.64	1.39	1	14
**People in household – neg**.	2.63	1.38	1	14
**People in household – pos**.	3.50	1.61	1	6
	**Female**	**Male**	**% female**	**% male**
**Female/male – all**	301	83	78.39	21.61
**Female/male – neg**.	297	81	78.57	21.43
**Female/male – pos**.	4	2	66.67	33.33
	**No**	**Yes**		
**Migration – all**	354	30		
**Migration – neg**.	348	30		
**Migration – pos**.	6	0		
	**no**	**Yes**	**% HCW**	
**Health Care Worker – all**	87	297	77.34	
**Health Care Worker – neg**.	87	291	76.98	
**Health Care Worker – pos**.	0	6	100.00	
	**No**	**Yes**	**No answer**	
**High-risk person in household – all**	292	90	2	
**High-risk person in household – neg**.	288	88	2	
**High-risk person in household – pos**.	4	2	0	
	**No**	**Yes**		
**Children in household – all**	225	155	4	
**Children in household – neg**.	223	151	4	
**Children in household – pos**.	2	4	0	
	**No**	**Yes**		
**Smoking – all**	318	65	1	
**Smoking – neg**.	312	65	1	
**Smoking – pos**.	6	0	0	

378 participants had a negative antibody test result (98.4%). Antibodies to the SARS-COV-2 virus were detected in 6 participants (1.6%). 4 had a positive PCR test. 2 participants (0.5%) went through the infection with the virus with minimal symptoms, and one even had a negative PCR test.

### Number of positive RT-PCR tests and comparison to regional reported prevalence

4 of 384 participants reported a positive PCR test result. The resulting estimate for the prevalence among healthcare workers in this study was 1.04% (95% CI 0.41–2.65%) and higher than the regional prevalence of PCR positives reported for the general population (0.204%, adjusted for age and sex) by a factor of 5.1 (95% CI 2.0–13.0). The difference was statistically significant with p=0.01 (after Bonferroni-Holm correction).

Antibody tests for this study were made available throughout June 2020. Antibodies can be detected 10–15 days after the onset of symptoms [9]. No currently positive cases were included in the study. Quarantine time in Germany was 14 days at the time of the study. Therefore, relevant comparable prevalence for the general population in the region was calculated based on the average of cases reported by the RKI for the first half of June 2020 (3,745 cases for a population of 2,055,774 in the Regierungsbezirk Detmold, *i.e*., 0.182% [Table 2]). The estimate for regional prevalence should be on the conservative side, as the selection of the greater region of the administrative district of Detmold delivers a higher prevalence than if only looking at the city of Bielefeld, in which the hospital is based (411 cases for a population of 334,195, *i.e*., 0.12%).

**Table 2:** Prevalence for hospital workers in the study compared to the general population.

Study prevalence vs. regional prevalence (PCR positive cases)	PCR+	Population	Percent	Factor
Prevalence general population in region (Ø 1–15 June 2020)	3,745	2.056 m	0.182%	-
Factor for age and sex adjustment	-	-	-	1.12
Expected cases in the study (adjusted for age and sex)	0.78 [95% CI 0–2.5]	384	0.204%	-
Observed cases in the study population	**4**	**384**	**1.04%**	-
Factor prevalence study population/general population	-	-	-	5.11*

* – p=0.001 (after Bonferroni-Holm correction).

Adjustment factors for age (adults of working age: 1.096) and sex (females 1.045, males 0.957) were calculated based on cases reported by the RKI for all of Germany (adults defined as an age group of 20–65 years, the female-to-male case ratios for age group 15–59) [6, 10, 11]. There were slight differences in the definitions of the groups due to the limited granularity of the data reported by the RKI. However, given the relatively small effect, this should not have any significant impact on the results.

### The ratio of positive antibody tests vs. positive PCR tests

Antibodies to the SARS-CoV-2 virus were found in 6 participants (1.6%, 95% CI 0.72–3.4%), yielding a ratio between seroprevalence and reported PCR positive prevalence of 1.5 (95% CI 1.1–3.5). This is significantly higher (p=0.011 after Bonferroni-Holm correction, p=0.005 without correction) than the expected value based only on PCR positive prevalence in the general population (after adjusting for age, sex, and test specificity: 0.404%). The number of previously undetected COVID-19 cases needs to be evaluated in the context of the hospital's testing policy, which gave participants easy access to PCR tests: 41.1% of the participants reported having been PCR-tested for SARS-CoV-2 (57.3% not tested, 1.6% did not reply).

The ratio found in this study was slightly lower than the ratios found in five other studies between April and September 2020, with over 17,000 participants ranging from 1.6 to 6.0. While the data on the various studies is not sufficient for statistical analysis, the comparison of the studies in Figure 1 shows that the highest ratios (4.5, 5.0, and 6.0) were seen in 3 studies in regions where the reported PCR prevalence was also highest. The ratio of 1.5 measured in this study is the lowest. This agrees with a relatively low overall prevalence (1.04% for the study population) and the fact that the study population had far easier access to PCR tests than the general population in Germany. This is due to the hospital's policy to offer PCR tests to all employees with symptoms, those working in high-risk areas, or who had contact with a COVID-19 case.

### Symptoms associated with COVID-19

14 different symptoms were asked about in the questionnaires. 11 of these showed significant odds ratios in the previous study by Streeck et al. in Gangelt (Germany) in April 2020 [12]. In this study, participants were asked whether they had experienced symptoms over the winter of 2019/2020. 72.4% of the participants suffered a cold between October 2019 and March 2020. Of these, 75.9% had one or several slight colds, and 24.1% had one or more heavy colds. When asked about the occurrence of symptoms that could be associated with a COVID-19 infection, 50% of the participants said they had noticed at least one of them in 2020. Of the participants who tested positive, 100% had noticed several such symptoms (2 only mild). Overall, the symptoms are nonspecific symptoms associated with numerous colds.

As a result, statistically significant odds ratios were found only for loss of smell (OR 53.0; 95% CI 9.06–310.04, p<0.0001) and loss of taste (OR 33.36, 95% CI 6.04–184.29, p<0.0001). Table 3 shows details and a comparison with the findings by Streeck et al. [12].

**Table 3:** Symptoms reported and odds ratios compared to Streeck et al.

Symptom	Hospital study					Gangelt study Streeck et al.			
	x w/symptoms					x w/symptoms			
	AB+	AB-	Odds ratio [0.95 CI]	P-value^(a)^	P-value^(b)^	AB+	AB-	Odds ratio [0.95 CI]	P-value
**Loss of smell**	50.0%	3.8%	53 [9.06; 310.04]	**0.00001**	**0.0013**	22.6%	1.4%	19.54 [9.03; 42.3]	**<0.001**
**Loss of taste**	50.0%	5.9%	33.36 [6.04; 184.29]	**0.0000**	**0.0065**	27.0%	1.9%	17.44 [8.71; 34.91]	**<0.001**
**Fever of 38°C**	16.7%	18.3%	2.02 [0.23; 17.83]	0.26	1	23.9%	6.0%	4.63 [2.7; 7.94]	**<0.001**
**Sweats and chills**	0.0%	15.6%	0.91 [0.05; 16.58]	0.53	1	29.2%	9.8%	3.74 [2.31; 6.06]	**<0.001**
**Fatigue**	33.3%	61.3%	1.16 [0.21; 6.41]	0.43	1	43.1%	19.0%	3.05 [2.01; 4.63]	**<0.001**
**Dry cough**	16.7%	41.9%	0.77 [0.09; 6.68]	0.59	1	51.4%	26.3%	2.77 [1.9; 4.06]	**<0.001**
**Muscle and joint ache**	33.3%	34.4%	2.45 [0.44; 13.68]	0.15	1	24.8%	11.6%	2.46 [1.49; 4.08]	**0.004**
**Chest tightness**	0.0%	14.0%	1.02 [0.06; 18.66]	0.49	1	13.9%	6.0%	2.38 [1.36; 4.16]	**0.014**
**Headache**	66.7%	54.8%	5.41 [0.98; 30]	0.03	1	35.8%	19.0%	2.28 [1.47; 3.54]	**0.002**
**Shortness of breath**	16.7%	21.0%	1.74 [0.2; 15.26]	0.31	1	8.7%	4.4%	2.08 [1.07; 4.06]	0.127
**Sore throat**	33.3%	67.2%	1.01 [0.18; 5.6]	0.49	1	31.1%	18.5%	1.96 [1.27; 3.02]	**0.014**
**Nose congestion**	33.3%	61.3%	1.16 [0.21; 6.41]	0.43	1	44.0%	26.2%	1.9 [1.27; 2.86]	**0.013**
**Other resp. symptoms**	0.0%	8.1%	1.8 [0.1; 33.48]	0.35	1	10.4%	7.1%	1.51 [0.82; 2.78]	0.556
**Diarrhea**	16.7%	16.7%	2.16 [0.25; 19.08]	0.24	1	n/a	n/a	n/a	n/a

(a) – unadjusted; ^(b)^ – adjusted for multiple testing with Bonferroni-Holm correction. Significant odds ratios in bold.

### Attitude, risk factors, and behavior during the first wave of the pandemic

93.5% of the participants found the measures to restrict contact during the first lockdown appropriate, 5% did not, and 1.5% did not reply. Support for the lockdown during the first wave of the pandemic in March/April 2020 was slightly higher than in the German population. This was measured by the German Federal Institute for Risk Assessment (BfR) during its weekly representative surveys using the same question used here, with agreement ranging from 92% on March 24, 2020, to 77% on April 28, 2020, at the end of the lockdown [13].

**Figure 2:**
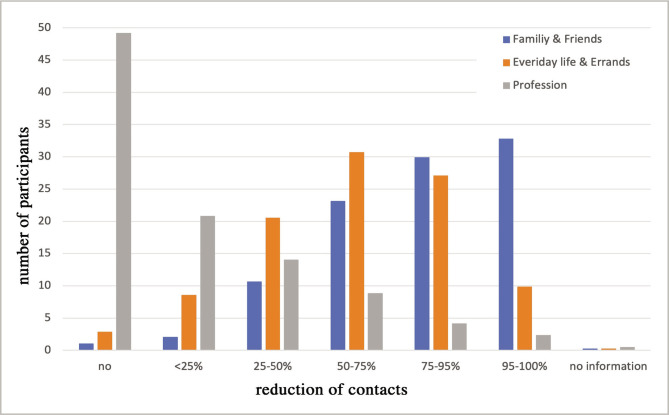
Reduction of contacts during the first lockdown (March/April 2020) differentiated between private and professional contacts.

During the first lockdown (March/April 2020), 93.8% of participants reported regular compliance with protective measures (mouth-nose mask, spacing, basic hygiene). A similar share of participants significantly reduced their private contacts amongst "family and friends" and slightly less in "everyday life and errands" (Figure 2). This was not the case for "profession". Accordingly, the number of "professional" contacts was significantly higher amongst participants with a medical profession (p<0.001).

When asked if further contact limitations above the level of the measures in April 2020 would be possible without significantly decreasing quality of life, only 8.1% answered with "yes" and 31.5% with "partly". 57.5% of the participants answered "no", and 2.9% did not answer. An analysis revealed no significant influencing parameter.

**Figure 3:**
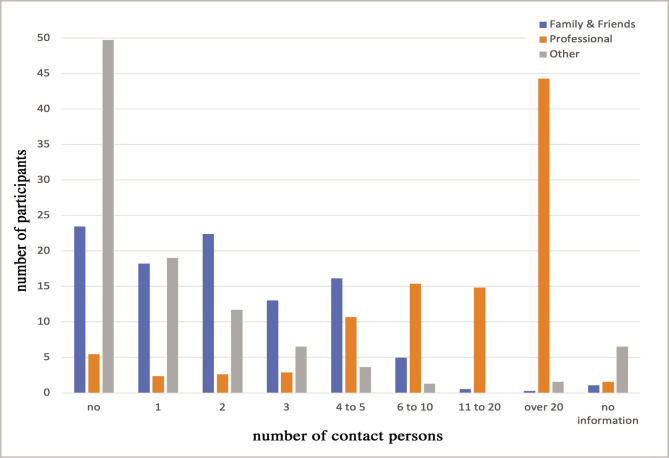
Number of different contact persons per week in April 2020.

Figure 3 shows the average number of different contact persons per week in April 2020 (lockdown) for "Family & Friends", "Profession" and "Other". There was a significant relationship between higher numbers of contacts in "Family & Friends" and attitudes of "not appropriate" on contact restriction measures (p=0.005).

While only 4 participants had a positive PCR test and 6 showed a positive antibody test, 60 participants (15.6%) reported already having quarantined themselves at some time during the first wave of the pandemic. The mean duration was 4.4 days (SD 4.8, range 1 to 21 days). Factors significantly favoring quarantine were a medical profession (p=0.021), a high number of professional contacts (p=0.045), and presence at work (p=0.015).

Because of existing symptoms, 80 participants (20.8%) reported contacting a doctor, and 22 (5.7%) participants contacted the regional health department. 41.1% of the participants reported having had a PCR test for SARS-CoV-2. 57.3% had not had a PCR test, and 1.6% did not answer. The only significant influence on having a SARS-CoV-2 test done was having contact with COVID-19-positive persons (p<0.001).

Looking at the risk factor "COVID-19 contacts", 5 participants reported having had COVID-19-positive contacts in the household. 43 participants had this in a private or professional environment (contact for more than 15 minutes and at a distance below 1.5 m). Of the 6 antibody-positive participants, only 1 had positive contact in the household, whereas 4 had contact in a private or professional environment.

17% of the participants reported that they currently smoked (16% cigarette, 1% e-cigarette, an average of 11 cigarettes/day). The participants indicated the following concomitant diseases as further health-specific risk factors (allergies 119, 70 cardiovascular diseases, 33 obesity, 31 chronic lung disease, 29 autoimmune diseases, and 6 diabetes mellitus). The sum of the secondary diagnoses had a significant influence on the daily behavior of the participants surveyed; thus, as the number of secondary diagnoses increased, the proportion of participants who did their own shopping decreased (p=0.002).

**Figure 4:**
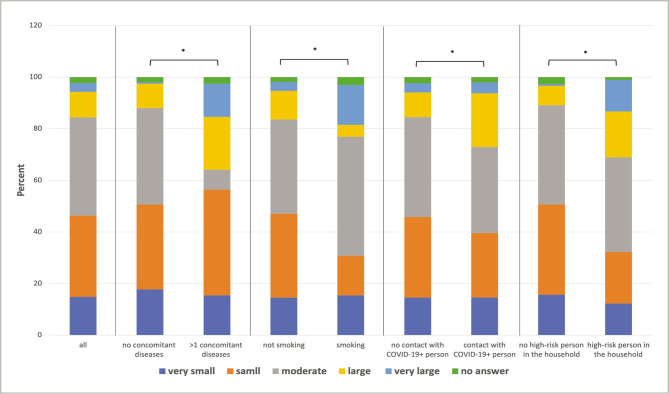
Expected personal health effects of a COVID-19 infection measured with a visual analogue scale. Results presented for the whole study population and selective sub-populations with significant influence. (* – p<0.05).

When asked about the expected personal health effects of a COVID-19 infection, 46.4% of the participants said "very small/small". 38% expected "moderate", and 13.3% expected "great/very great" health effects. 2.3% of the participants did not answer (Figure 4). Factors with a significant impact on the expected personal health impact of a SARS-COV-2 infection include age, having a high-risk person in the household, a concomitant illness, contact with a COVID-19-positive person, and smoking (all p<0.001). All these factors lead to a shift in the estimated health impact towards "very great".

**Figure 5:**
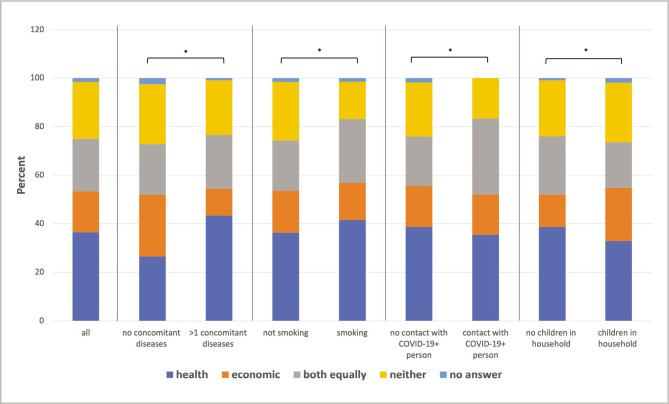
Concerns about health or economic effects for the whole study population and selective sub-populations with significant influence. (* – p<0.05).

When asked if the participants were more concerned with the health or the economic effects of the corona pandemic, the majority (36.5%) answered health effects, 17% economic effects, 21.6% both equally, 23.4% neither, and 1.5% did not answer (Figure 4). Parameters that significantly influenced this statement were: concomitant diseases (p=0.009), smoking (p<0.001), contact with a COVID-19-positive person (p<0.001), and children in the household (p=0.003). The first 3 parameters lead to prioritization of health or both effects, while children in the household lead to prioritization of economic effects (Figure 5).

## DISCUSSION

The present study combined a SARS-CoV-2 antibody test with a questionnaire to investigate the epidemiology in a risk population (health care workers) and to inquire about potential risk factors, attitudes, and behavior of the participants during the first lockdown in March/April 2020.

In this study, a cross-section of the working population at a maximum care hospital was surveyed, including 76.8% healthcare workers.

### Increased risk of COVID-19 for health care workers

4 participants reported a positive PCR test. The resulting estimate for the prevalence amongst health care workers of 1.04% was significantly higher, by a factor of 5.5, than the regional PCR-positive prevalence reported for the general population. These results agree with earlier studies, which found that health care workers are a population at risk for a SARS-CoV-2 infection, especially in regions with a high incidence [14–16]. Nguyen et al. found a factor of 3.4 for the PCR frequency amongst healthcare workers and the general population during the first pandemic wave in March/April 2020 in a study on healthcare workers in the US and the UK [17]. Our analysis of officially reported PCR cases by the RKI for Germany showed a factor of 3.1.

Our data suggest that healthcare workers may be at higher risk of infection. Therefore, a discussion about prioritizing vaccination makes sense. It also supports the policy to make SARS-CoV-2 testing more available to hospital workers.

### Additional 50% of undetected COVID-19 cases with antibody testing vs. PCR testing

The ratio of seroprevalence to PCR prevalence found in this study of 1.5 was slightly lower than that in other seroprevalence studies in Germany for the same period after the first wave. We hypothesize that easier access to SARS-CoV-2 tests reduces the number of unreported cases and that in high-prevalence situations, when testing is limited, the share of unreported cases increases.

### Many symptoms associated with COVID-19 are relatively unspecific

Symptoms associated with COVID-19 also were widely prevalent in the non-COVID-19-positive population. In particular, during the winter season, when many people have other symptomatic respiratory infections, most symptoms associated with COVID-19 do not have a strong predictive value. The exceptions are loss of smell and loss of taste. This result agrees with Iversen et al., who showed loss of smell or taste to be those symptoms with the strongest association to a positive SARS-CoV-2 antibody test [16]. 32.39% of their seropositive participants showed one of these symptoms, compared with 50% of the seropositive participants in our cohort. This also agrees with the review by Zahra et al., who listed studies on the prevalence of taste or smell disorders, which ranged from 34–84% of seropositive individuals [18].

In this study, all participants with a positive antibody test reported several symptoms. These results are in contrast to those of Yakamishi et al., who reported 14% of asymptomatic persons during the outbreak on a cruise ship diagnosed using PCR [19]. In a recent review by Gao et al., incidences of asymptomatic COVID-19-positive individuals (PCR test) ranged from 1.6–56.5% [3].

### Health workers were supportive of lockdown measures and showed consistent behavior in protecting themselves in their private life

Health workers were largely supportive of non-pharmaceutical interventions and more supportive of lockdown measures than the general population. By and large, they also followed the rules and recommendations for reducing private contacts during the lockdown. Compliance with hygiene and distancing rules to reduce the spread of SARS-CoV-2 was very strong in our collective. Zhang et al. reported that 89.7% of 1,357 healthcare workers surveyed followed the recommended rules [20]. They identified gender, knowledge, and attitude towards COVID-19 as significant factors influencing hand hygiene. Influencing factors for wearing a face mask were gender, parental health, and attitude toward COVID-19. Measures of social distancing were influenced by parental health and attitude towards COVID-19. In our study, we found the behavior to correlate the strongest with the attitude towards political measures and perceived individual risk factors. This agrees with other studies on the effects and predictors of compliance with COVID-19 rules and recommendations. Hills et al. found that the attitude towards social distancing rules significantly influenced behavior and compliance with rules and recommendations [21]. Choma et al. found that individual risk factors, particularly individual health risks, had a significant impact on compliance with COVID-19 rules and recommendations [22].

From this, we draw two conclusions: health workers are largely similar in their attitudes to the general population, although they tend to show greater compliance. Compliance can at least partially be explained by rational behavior based on personal risks. 46.4% of the respondents in our cohort reported that they rated the personal health consequences of COVID-19 as "very small or small". This may be due to the mild course of the first wave of the pandemic in most parts of Germany. However, increasing age, concomitant diseases, having persons at risk in the household, contact with someone infected with COVID-19, and smoking led to a shift in the assessment to "great and very great". It is known from the literature that certain concomitant diseases can lead to a more severe course or fatal outcome of COVID-19 disease. Hypertension, diabetes and cardiovascular disease, tumors, chronic kidney disease, and respiratory disease have been identified so far [23–25]. The impact of these diseases on personal behavior shows that this information is also considered in the individual decisions made by the participants in this study.

In addition, we believe that individual attitudes towards rules and regulations on COVID-19 affect compliance. Therefore, one goal of public policy-making should be to maintain the support of non-pharmaceutical measures at a high level in order to keep actual compliance up.

### Limitations of the study

One limitation was that the response rate was lower than expected (15.1%), thus lowering the overall statistical power. This might have been because the questionnaire was relatively long, and for reasons of data protection, the questionnaire could only be sent to participants after they had already been tested for antibodies. Participants were self-selected, which may have introduced bias into the study cohort. However, we did a cross-section of the employees at the hospital. The positive rate of SARS-COV-2 antibody testing was very low, although this was consistent with what occurred during the first wave in most parts of Germany (except hot spots).

## CONCLUSION

Our data suggest that healthcare workers may be at higher risk of infection. They also support offering increased SARS-CoV-2 testing to hospital workers. It is concluded that easier access to SARS-CoV-2 testing reduces the number of unreported cases. Furthermore, individual attitudes toward rules and regulations on COVID-19 critically influence compliance. Thus, one goal of public policy should be to maintain high levels of support for non-pharmaceutical interventions to keep actual compliance high.

## Supplementary Material



## Data Availability

Further data is available from the corresponding author on reasonable request.

## Personal thanks

We thank Odile Sauzet for assistance with the statistical analysis.
