# Supplementary material for: COVID-19 after the first wave of the pandemic among employees from a German university hospital: prevalence and questionnaire data
Source: J Med Life. 2022 Sep;15(9):1119–28. doi: 10.25122/jml-2022-0126 (PMC9635233; doi:10.25122/jml-2022-0126)
Supplement: Supplementary file 1 [file JMedLife-15-1119-s001.pdf]

COVID-19: Antikörpermessung an ausgewählten Kollektiven zur Untersuchung der Infektionsepidemiologie

Teilnehmer-Nr.

____
Nachname

____
Vorname

☐☐.☐☐.☐☐☐☐
Geburtsdatum (TT.MM.JJJJ)

☐☐☐☐☐
Postleitzahl

____
Telefonnummer

____
E-Mail-Adresse

Hinweise zum Ausfüllen des Fragebogens

→

Rückseite

Supplementary Figure 1. Questionnaire applied in the study.

Deutsches Institut für Orthopädie Osteopathie und Sportmedizin

EVANGELISCHES KLINIKUM Bethel

- Bitte beachten Sie, dass es beim Fragebogen eine Rückseite gibt.
- Wenn Sie in der Vergangenheit per Abstrichtest positiv auf COVID-19 getestet wurden und damit die Frage 11 mit „ja“ beantworten, so beantworten Sie bitte die weiteren Detailfragen auf dem Extrablatt. Bitte füllen Sie dieses Extrablatt nur aus, wenn Sie schon einmal positiv auf COVID-19 getestet worden sind!
- Wenn Sie eine Frage nicht beantworten möchten oder die Antwortmöglichkeiten auf Sie nicht zutreffen, so kreuzen Sie bitte keine Antwort an und lassen das entsprechende Feld leer.
- Wenn keine Auswahlmöglichkeiten angegeben sind, schreiben Sie bitte Ihre Antwort in das vorgesehene Feld (z.B. eine Zahl bei Frage zum $_{F1}$Alter oder Freitext bei der Frage zu Ihrer $_{F3}$Muttersprache).
- Bei Fragen zu Ihren Kontakten möchten wir die Reaktion auf COVID-19-Maßnahmen erfahren. Beziehen Sie Ihre Antworten daher immer auf die Zeit nach den bundes- oder landesweiten Maßnahmen gegen COVID-19 ab Mitte März und vor den Lockerungen ab Mai 2020 (also insb. April 2020).

Vielen Dank für Ihre Teilnahme!

Supplementary Figure 1. Continued.

Deutsches Institut für Orthopädie Osteopathie und Sportmedizin

EVANGELISCHES KLINIKUM Bethel

F0 Datum (TT.MM.JJJJ)	

Teilnehmer-Nr.

Ergebnis Antikörper-Test	

FRAGEN ZUM PERSÖNLICHEN HINTERGRUND

F1 Alter	Jahre
F2 Geschlecht	□ männlich □ weiblich □ divers
F3 Was ist Ihre Muttersprache?	
F4 Haben Sie einen Migrationshintergrund?	□ ja □ nein
F5 Was ist Ihr höchster Bildungsabschluss? □ keiner<br>□ Haupt-/Volksschule □ Realschule/Mittlere Reife □ Gymnasium/Abitur<br>□ Abgeschl. Ausbildung □ FH-Diplom/Bachelor □ Uni-Diplom/Master/Magister □ Promotion	
F6 Hatten Sie einen Auslandsaufenthalt seit Dezember 2019?	□ ja, in □ nein
F7 Wie beurteilen Sie Maßnahmen zum Kontaktverbot, die zur Eindämmung der Ausbreitung des neuartigen Coronavirus getroffen wurden?<br>□ angemessen □ nicht angemessen	
F8 **Berufstätigkeit**	
F8.1 Welchen Beruf haben Sie erlernt?	□ keinen
F8.2 Welche berufliche Tätigkeit üben Sie aus? (im April 2020)	□ keine
F8.3 Umfang der Berufstätigkeit (wenn Sie Nebentätigkeiten haben, antworten Sie bitte nur für die Haupttätigkeit)<br>□ keine □ Teilzeit, zu ______ % □ Nur Minijob oder ähnliches<br>□ Vollzeit □ Kurzarbeit, reduziert auf ______ %	
F8.4 Art der Berufstätigkeit<br>□ öffentlicher Bereich mit vielen Kontakten (z.B. Gesundheitswesen / Supermarkt /Dienstleistung)<br>□ Arbeitsbereich mit wenigen Kontakten (z.B. Büro / Bauwesen / Kanzlei etc.)<br>□ Arbeitsbereich ohne Kontakte	
F8.5 Ort der Berufstätigkeit im April 2020<br>□ keine □ vollständig im Home Office<br>□ teils Home Office / teils am Arbeitsplatz □ vollständig am Arbeitsplatz	
F9 **Fragen zur Wohnsituation und Haushaltsgröße**	
F9.1 Wie würden Sie Ihre Wohnsituation beschreiben?	□ städtisch □ ländlich
F9.2 Größe Ihrer Wohnung / Ihres Hauses	m^2
F9.3 Anzahl der Personen in Ihrem Haushalt	Personen
F9.4 Leben in Ihrem Haushalt andere Personen, die nach Ihrer Auffassung ein besonderes Risiko bei COVID-19 hätten (Vorerkrankungen, Alter)?	□ ja □ nein
F9.5 Leben in Ihrem Haushalt Kinder?	□ ja □ nein

	Alter (in Jahren)	*Wenn das Kind Kita oder Schule besucht, bitte zutreffendes ankreuzen*		
		Kita	Grundschule	Andere Schule
Kind 1		□	□	□
Kind 2		□	□	□
Kind 3		□	□	□
Weitere		□	□	□

Supplementary Figure 1. Continued.

FRAGEN ZUR GESUNDHEIT

F10 Haben Sie in diesem Jahr jemals Symptome an sich bemerkt, die oft COVID-19 zugeschrieben werden? □ ja □ nein

F10.1 Wenn ja: Welche Symptome haben Sie an sich bemerkt? (Mehrere Antworten möglich)

□ Fieber über 38°C	□ Halsschmerzen
□ Schüttelfrost	□ Kopfschmerzen
□ Gliederschmerzen	□ Trockener Husten
□ Verstopfte Nase / Schnupfen	□ Kurzatmigkeit
□ Verlust von Geruchssinn	□ Engegefühl in der Brust
□ Verlust von Geschmacksinn	□ Andere Atembeschwerden
□ Erschöpfung oder Abgeschlagenheit	□ Durchfall

F10.2 Haben Sie sich jemals selbst unter Quarantäne gestellt? □ ja □ nein

F10.2.1 Wenn ja: Wieviel Tage nach den ersten Symptomen? _____ Tage

F10.3 Haben Sie einen Arzt oder das Gesundheitsamt kontaktiert?

F10.3.1 Arzt □ nein □ ja Wieviele Tage nach ersten Symptomen? ____

F10.3.2 Gesundheitsamt: □ nein □ ja Wieviele Tage nach ersten Symptomen? ____

**F11** Wurde bei Ihnen bereits ein COVID-19 Test durchgeführt?	□ ja □ nein
**F12** Wurde bei Ihnen eine COVID-19 Erkrankung diagnostiziert? **(Wenn ja, dann bitte Detailfragen auf Extrablatt beantworten)**	□ ja □ nein
**F13** Besteht/bestand eine COVID-19 Erkrankung in Ihrem Haushalt?	□ ja □ nein
**F14** Besteht/bestand eine nachgewiesene COVID-19 Erkrankung im nahen familiären oder beruflichen Umfeld (Kontakt >15min mit Abstand <1,5m)?	□ ja □ nein

F15 Sind Sie Raucher?

□ ja, Zigarette: ______ Stück pro Tag □ ja, E-Zigarette □ nein

F16 Welche Krankheiten haben Sie? (mehrere Antworten möglich)

□ Diabetes (Typ I oder Typ II)	□ Krebs
□ Chronische Lungenerkrankung (COPD, Asthma)	□ Allergien
□ starkes Übergewicht / Adipositas	□ Autoimmunerkrankung
□ Herz- oder Kreislauferkrankung (Bluthochdruck, Herzinsuffizienz)	□ Andere chronische Erkrankung(en): ____________________________________

F17 Angenommen, Sie hätten COVID-19: Wie groß oder klein schätzen Sie die gesundheitlichen Auswirkungen davon für Sie persönlich ein? (Antwortskala: 1 „sehr klein“ bis 5 „sehr groß“)

sehr klein	1	2	3	4	5	sehr groß
	□	□	□	□	□	

**F18** Waren Sie im Winterhalbjahr (Okt 2019 – März 2020) erkältet?	□ ja □ nein

Supplementary Figure 1. Continued.

F18.1 Wie oft waren Sie im Winterhalbjahr (Okt 2019 – März 2020) etwa erkältet?
□ Einmal leicht (leichter Schnupfen/Husten, kein Fieber)
□ Einmal schwer (Schnupfen/Husten, mehr als 1 Tag Fieber)
□ 2-3mal leicht (leichter Schnupfen/Husten, kein Fieber)
□ 2-3mal, davon mind. 1mal schwer (Schnupfen/Husten, mehr als 1 Tag Fieber)
□ 4mal und öfter leicht (leichter Schnupfen/Husten, kein Fieber)
□ 4mal und öfter, davon mind. 1mal schwer (Schnupfen/Husten, mehr als 1 Tag Fieber)

F19 Was denken Sie, was betrifft Sie stärker: die gesundheitlichen oder die wirtschaftlichen Auswirkungen des neuartigen Coronavirus?
□ gesundheitlichen Auswirkungen
□ beide gleichermaßen
□ wirtschaftlichen Auswirkungen
□ keine von beiden

FRAGEN ZU PRIVATEN UND BERUFLICHEN KONTAKTEN

F20 Während der Einschränkungen durch COVID-19 Maßnahmen (insb. April 2020)
□ habe ich selbst eingekauft
□ war ich in ärztlicher Behandlung
□ hatte ich privat häufig Kontakt zu Personen außerhalb der Familie
□ war mindestens eins meiner Kinder in der Notbetreuung (Kita, Schule)
□ habe ich mich regelhaft geschützt (Mundschutz, mind. 1,5m Abstand)

F21 Haben Sie die Anzahl Ihrer Kontakte außerhalb Ihres Haushalts im April 2020 reduziert ggü. der Zeit vor COVID-19? (Zu Kontakten gehören auch Kontakten bei denen Sie auf Abstand (weniger als 1,5m) geblieben sind oder eine Maske getragen haben).

F21.1 Privat - Freunde und Familie (abgesehen von Personen Ihres Haushalts)
□ Nein
□ Kaum (weniger als 25% Reduktion)
□ Mittel (25-50% Reduktion)
□ Stark (50-75% Reduktion)
□ Sehr stark (75-95% Reduktion)
□ Fast komplett (95-100%)

F21.2 Privat - Alltag und Besorgungen (z.B. Einkäufe, Friseurbesuche, Arztbesuche)
□ Nein
□ Kaum (weniger als 25% Reduktion)
□ Mittel (25-50% Reduktion)
□ Stark (50-75% Reduktion)
□ Sehr stark (75-95% Reduktion)
□ Fast komplett (95-100%)

F21.3 Beruflich
□ Nein
□ Kaum (weniger als 25% Reduktion)
□ Mittel (25-50% Reduktion)
□ Stark (50-75% Reduktion)
□ Sehr stark (75-95% Reduktion)
□ Fast komplett (95-100%)

Supplementary Figure 1. Continued.

F21.4 Wenn Ihre beruflichen Kontakte reduziert wurden, wodurch ist das hauptsächlich erfolgt?

□ Aktive Maßnahmen des Arbeitgebers (z.B. Home Office)
□ Reduktion / Wegfall des Geschäfts (z.B. keine Kunden mehr oder weniger Kunden)
□ Teils/Teils □ Keine Reduktion oder nicht zutreffend

F22 Mit wieviel verschiedenen Personen außerhalb Ihres Haushalts hatten Sie innerhalb einer durchschnittlichen Woche im April 2020 Kontakt (entweder mit Abstand von weniger als 1,5m für 15 Minuten oder gemeinsamer Aufenthalt in einem geschlossenen Raum für mehr als 30 Minuten)?

F22.1 ***Freunde und Familie***	F22.2 ***Berufliche Kontakte***	F22.3 ***Sonstige (z.B. Arzt)***
□ keine	□ keine	□ keine
□ 1	□ 1	□ 1
□ 2	□ 2	□ 2
□ 3	□ 3	□ 3
□ 4-5	□ 4-5	□ 4-5
□ 6-10	□ 6-10	□ 6-10
□ 11-20	□ 11-20	□ 11-20
□ über 20	□ über 20	□ über 20

F23 Wäre eine weitere Reduktion der Kontakte über das, was Sie im April 2020 gemacht haben, für Sie möglich, ohne wesentliche Einschränkung der Lebensqualität?
□ ja □ teilweise □ nein

Maßnahmen des Arbeitgebers *(bei Selbstständigen: Ihre eigenen Maßnahmen)*

F24 Welche Maßnahmen führt Ihr Arbeitgeber für Sie durch zur Reduktion der Kontakthäufigkeit / der Kontaktgruppen (insb. April 2020)?
□ ausschließlich Home Office
□ vermehrt Home Office
□ kein Home Office → **F24.1** Wäre Arbeit vom Home Office Ihrer Ansicht nach möglich?
□ ja □ teilweise □ nein

F25 Wenn Sie noch beim Arbeitgeber / beim Kunden vor Ort arbeiten, gibt es Regelungen dazu, wie Mitarbeiter ggf. in definierten Gruppen zusammenarbeiten?
□ Ausschließlich Arbeit in kleineren gleichbleibenden Gruppen (z.B. A/B-Besetzung)
□ Teilweise Arbeit in kleineren gleichbleibenden Gruppen (kann bedarfsbezogen wechseln)
□ Keine Einteilung in Gruppen
□ Nicht anwendbar

F25.1 Wäre Arbeit in kleineren gleichbleibenden Gruppen Ihrer Ansicht nach möglich?
□ ja □ teilweise □ nein

F26 Reduktion Kunden-/Zuliefererkontakte (nur aktive Maßnahmen Ihres Arbeitgebers, Reduktion aufgrund Wegfall/Reduktion des Geschäfts nicht berücksichtigt)
□ Keine aktive Reduktion
□ Kaum aktive Reduktion
□ Keine physischen Kontakte mehr
□ Starke aktive Reduktion der physischen Kontakte (durch klare Regeln, welche Kontakte nicht mehr erfolgen sollen)
□ Keine Kontakte mehr mit Abstand von weniger als 1,5m

F26.1 Wäre eine Reduktion der Kunden-/Zuliefererkontakte Ihrer Ansicht nach möglich?
□ ja □ teilweise □ nein

Supplementary Figure 1. Continued.

DiOOS
Deutsches Institut für Orthopädie Osteopathie und Sportmedizin

EVANGELISCHES KLINIKUM Bethel

Detailfragen

Teilnehmer-Nr.

Nur für Personen, bei denen in der Vergangenheit eine COVID-19 Erkrankung diagnostiziert wurde.

Zu Frage F12: Wenn bei Ihnen eine COVID-19 Erkrankung diagnostiziert wurde

F12.1 Wann wurde der Testabstrich genommen? (Datum (TT.MM.JJJJ))?	______.______.____________
F12.2 Hatten Sie COVID-19 spezifische Symptome? (z.B. Atembeschwerden, Husten, Fieber, Ermüdung, Geruchs- oder Geschmacksverlust, Schüttelfrost, Erschöpfung oder Abgeschlagenheit)	□ ja, starke Symptome<br>□ ja, milde Symptome<br>□ nein, keine Symptome
F12.2.1 Erste Symptome am (Datum (TT.MM.JJJJ)):	______.______.____________
F12.2.2 Letzte Symptome am (Datum (TT.MM.JJJJ)):	______.______.____________
F12.3 Wurden Sie wegen COVID-19 stationär im Krankenhaus behandelt?	□ ja □ nein
F12.3.1 Erste Aufnahme im Krankenhaus (Datum (TT.MM.JJJJ)):	______.______.____________
F12.3.2 Letzte Entlassung aus dem Krankenhaus (Datum (TT.MM.JJJJ)):	______.______.____________
F12.3.3 Wurden Sie im Krankenhaus künstlich beatmet?	□ ja □ nein
F12.3.3.1 Wie lange (Anzahl Tage) wurden Sie künstlich beatmet?	__________ Tage

Supplementary Figure 1. Continued.

Supplementary Table 1. Summary of the chi-square test results. Significant results are printed in bold letters.

	Acceptance of measures	COVID-19 test	Quarantine	COVID-19 symptoms	Number of contacts		
					Family & friends	Professional	Everyday life & errands
**Age**	0.797	0.666	0.082	0.473	0.214	0.621	0.318
**Sex**	0.843	0.093	0.991	0.385	0.971	0.550	0.336
**Migration**	0.699	0.199	0.377	0.254	0.930	0.645	0.868
**Medical profession**	0.349	0.233	**0.021**	0.183	0.867	**<0.001**	0.084
**Education degree**	0.100	0.949	0.707	**0.005**	-	-	-
**Professional contacts**	0.986	0.862	**0.045**	0.488	0.985	**<0.001**	0.840
**Workplace**	0.939	0.882	**0.015**	0.285	-	-	-
**People in household**	0.942	0.262	0.309	0.060	-	-	-
**High-risk person in household**	0.648	0.677	0.646	0.316	-	-	-
**Children in household**	0.560	0.725	0.411	0.766	0.60	0.602	0.760
**Smoking**	0.540	**<0.001**	0.826	0.191	-	-	-
**Contact to COVID-19 pos.**	0.922	**<0.001**	0.743	0.571	0.284	0.962	0.894
**Sum of secondary diagnoses**	0.582	0.239	0.072	**0.044**	0.996	**0.160**	0.176
